# BanglaMix: a dataset of token-level annotated Bengali-English code-mixed social media comments with Bengali transliteration

**DOI:** 10.1016/j.dib.2026.113317

**Published:** 2026-10-07

**Authors:** Sabbir Mahmud Afridi, Abir Chakraborty Partha, Md. Mosaddek Khan

**Affiliations:** University of Dhaka, Dhaka 1000, Bangladesh

**Keywords:** Bengali-English code-mixing, Code-switching, Transliteration, Word-level language identification, Low-resource NLP, Social media text, Banglish

## Abstract

This article describes BanglaMix, a dataset of 12,576 Bengali-English code-mixed social media comments collected from a publicly available video-sharing platform (YouTube) using its public Data API. Each record pairs the original (raw) comment text with a lightly cleaned version, a Bengali-script (Unicode) transliteration of that text, and a word-level, token-by-token language annotation using five labels (BAN, ENG, ROM_BAN, OTHER, UNK). Each comment is additionally characterised by three comment-level attributes: script type (latin, bengali, mixed, or other), code-mix type (an eight-category label describing which languages and scripts co-occur in the comment, e.g., monolingual_BAN, ENG+ROM_BAN, BAN+ENG), and a boolean flag indicating whether the comment is likely to be code-mixed. All annotations, including the Bengali transliterations and the token-level language labels, were produced by a single primary annotator (the author); an inter-annotator reliability study in which a second bilingual annotator independently re-labelled a random 500-comment sub-sample found substantial agreement at both the token level (Cohen's kappa = 0.74) and comment level (Cohen's kappa = 0.67). Comments were retained as anonymous text records identified only by an alphanumeric comment identifier; no usernames, channel names, or other personal identifiers were stored, and records that had retained a public @-mention of another commenter were removed before release. Across the dataset, 57.3% of comments are written primarily in Latin script, 28.4% in Bengali script, and 13.6% in mixed script; 36.8% of comments are flagged as likely code-mixed. At the token level, of 120,866 labelled tokens, 44.7% are tagged as Bengali (BAN), 23.5% as English (ENG), and 22.1% as Romanized Bengali (ROM_BAN). Comments are short on average (median 6 words; mean 9.61 words per comment) and exhibit no duplicate source texts. The dataset is intended for reuse in word-level language identification, Bengali back-transliteration, and other Bengali-English code-mixed natural language processing tasks, for which large, token-annotated, naturalistic social media resources remain scarce.

## Highlights

• 12,576 Bengali-English comments with raw text, cleaned text, transliteration, and token labels.
• Word-level tags (BAN, ENG, ROM_BAN, OTHER, UNK) cover 120,866 tokens across all comments.
• First public resource pairing Bengali back-transliteration with 5-way token language tags.
• Comment-level script-type and code-mix-type labels support code-mixing-aware model evaluation.
• Silver-standard labels, validated by a 500-comment second-annotator reliability study (token-level kappa = 0.74; comment-level kappa = 0.67), with remaining limitations disclosed in full.

## Graphical abstract

**Figure:**
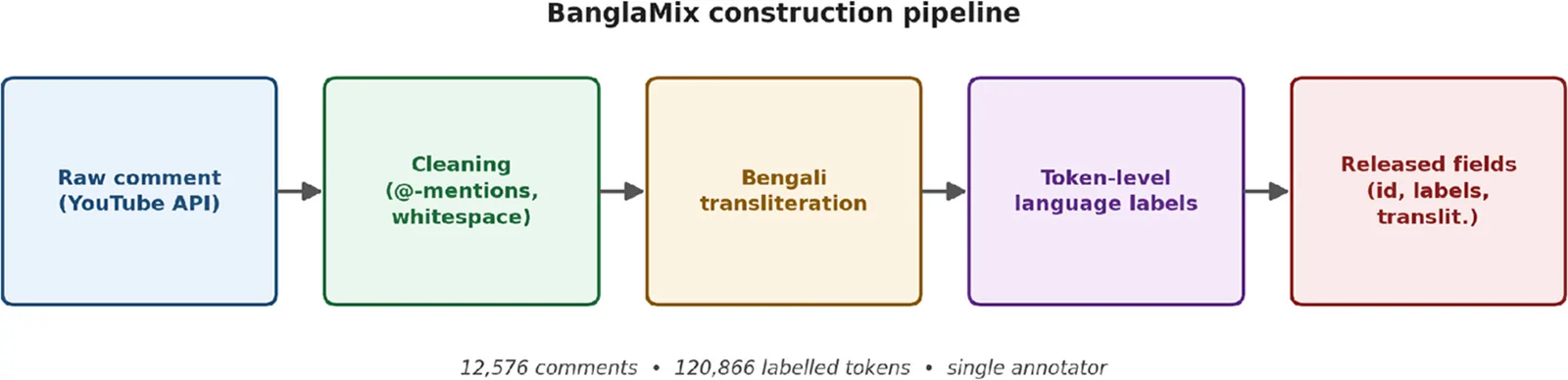

Specifications Table

**Table:** 

Subject	Computer Science
Specific subject area	Natural language processing of Bengali-English code-mixed social media text; word-level language identification and Bengali transliteration.
Type of data	Table (CSV file). Text: cleaned comments, Bengali-script transliterations, token-level language-tagged sequences, released directly. Raw and cleaned comment text obtainable via an accompanying re-hydration script keyed on the released comment ID (see Data Accessibility and Ethics Statement).
Data collection	Comments were collected from publicly available YouTube videos using the YouTube Data API v3 (commentThreads.list, playlistItems.list, and search.list endpoints), via a channel-based and/or search-based collection script. Comments authored by a video’s own channel/uploader were excluded at collection time; no length, spam, or language pre-filter was applied. Each record pairs a raw comment with a cleaned version (leading @-mentions removed; line breaks and repeated whitespace collapsed to single spaces), a Bengali-script transliteration, word-level language labels (BAN/ENG/ROM_BAN/OTHER/UNK), and comment-level script-type, code-mix-type, and likely-code-mixed tags, produced by a single primary annotator and validated by a second-annotator inter-annotator reliability study on a 500-comment sub-sample (token-level kappa = 0.74; comment-level kappa = 0.67). No usernames or other personal identifiers were stored beyond the platform-native comment ID (see Ethics Statement).
Data source location	Geographic and linguistic provenance was established during data collection using two complementary strategies. First, videos retrieved through the YouTube Data API were filtered using regionCode="BD" and relevanceLanguage="bn" to prioritize content associated with Bangladesh and the Bengali language. Second, the channel-based collection used a manually curated list of Bangladesh-based or predominantly Bangla-language YouTube creators selected by the author. To verify the effectiveness of this sampling strategy, a post-hoc manual audit was conducted on a random sample of 200 source channels. A channel was classified as Bangladesh-based when its YouTube About page indicated Bangladesh as the channel location or when its published content, descriptions, and community posts were predominantly in Bangla and targeted a Bangladeshi audience. Based on this audit, 184 of 200 channels (92%) met the Bangladesh-based criterion. All classifications were performed manually by the author.
Data accessibility	**Repository name:** Zenodo **Data identification number:**https://doi.org/10.5281/zenodo.22086156 **Direct URL to data:**https://zenodo.org/records/22086156 **Instructions for accessing these data:** The BanglaMix dataset is openly available through Zenodo at https://doi.org/10.5281/zenodo.22086156.
	
	The dataset can be downloaded from the Files section of the record as dataset_final.csv. The complete data-collection and reproducibility code is available in the accompanying GitHub repository: https://github.com/smahmuddz/BanglaMix. The repository contains rehydrate.py, which re-fetches currently available YouTube comment text using the comment_id values in the dataset and the researcher's own YouTube Data API credentials. The repository also provides requirements.txt and usage instructions in README.md. Comments that are no longer available through the YouTube API cannot be rehydrated. No registration or special permission is required to access the publicly available Zenodo record or GitHub repository. The complete list of the 56 seed queries used for the search-based collection is also included in the Zenodo record alongside the dataset, so that the search-based sampling frame can be reproduced exactly.
Related research article	None.

## 1 Value of the data

• These data provide a token-level, language-tagged parallel resource for Bengali-English code-mixed text drawn from naturalistic social media comments, pairing cleaned text, a Bengali-script transliteration, and word-level BAN/ENG/ROM_BAN/OTHER/UNK labels for the same utterance, with raw text obtainable via the accompanying re-hydration script. Large annotated resources of this kind remain scarce for Bengali, a widely spoken but comparatively low-resource language in NLP.
• Researchers working on word-level language identification for code-mixed South Asian languages can reuse these data to train or evaluate token-level classifiers, extending prior work developed mainly for other language pairs in the region.
• The paired cleaned_text / bengali_transliteration fields constitute parallel Romanized-to-Bengali transliteration examples that can be reused to train or benchmark back-transliteration systems.
• The comment-level script_type and code_mix_type annotations allow researchers to filter or stratify the data by the degree and form of code-mixing present, supporting downstream tasks such as sentiment analysis, offensive-language detection, or code-mixing-aware model evaluation once combined with additional task-specific labels.
• Because the data were drawn directly from publicly posted social media comments rather than being synthetically generated or elicited under laboratory conditions, they capture naturalistic patterns of Bengali-English code-mixing as they occur in real social media discourse.
• The raw_text and cleaned_text fields, obtainable via the accompanying re-hydration script, allow other researchers to apply their own text-normalisation pipelines to the original comment text rather than relying solely on the cleaning steps applied here.

## 2 Background

Bengali-English code-mixing (``Banglish'') is common in informal digital communication, including social media comments, yet large, token-annotated datasets that combine word-level language identification with Bengali-script transliteration remain limited relative to other South Asian code-mixed language pairs [[1], [2], [3], [4]], despite earlier linguistic analysis of the English-Bengali code-mixing phenomenon itself [5]. Prior work has produced word-level language-identification resources and shared tasks for Hindi-English and other Indian code-mixed pairs [2,3,[6], [7], [8]], cross-corpus comparisons of code-switching levels [9], sentiment-oriented Bengali-English and Dravidian code-mixed corpora sourced from social media and e-commerce platforms [10,11], and monolingual Bangla transliteration benchmarks [12,13]. This dataset was compiled to provide a comment-level and token-level annotated resource specifically pairing raw and cleaned Bengali-English code-mixed social media comments with their Bengali-script transliteration and word-level language tags, produced by a single annotator.

This data article is not related to a previously published research article by the authors.

## 3 Comparison with Existing Datasets

Table 1 situates BanglaMix relative to the closest prior code-mixed and Bengali-related resources cited above. The comparison highlights three main differentiators. First, BanglaMix is, to the author's knowledge, the first publicly released Bengali-English resource to combine word-level language identification with a Bengali-script transliteration for every comment: existing Bengali-English corpora such as BnSentMix [11] provide only comment-level sentiment labels, and the transliteration benchmark BanglaTLit [12] provides Romanized-to-Bangla pairs without accompanying code-mixed social media comments or language tags. Second, unlike the word-level LID corpora developed for Hindi-English and other Indian language pairs [[1], [2], [3], [4],^6^,^7^] and the Dravidian-English resources [8,10], which do not cover Bengali, BanglaMix supplies a 5-way token-level annotation (BAN, ENG, ROM_BAN, OTHER, UNK) specifically for Bengali-English code-mixing. Third, each comment in BanglaMix additionally carries comment-level script-type and code-mix-type labels, allowing the data to be filtered or stratified by the degree and form of code-mixing present, a layer of annotation not provided by the sentiment- or offensive-language-oriented datasets in Table 1. The comparison in Table 1 is drawn at the level of dataset design, scale, and annotation features; it is not a record-level overlap analysis, and no formal check for shared records between BanglaMix and these resources was performed (see Limitations).

**Table 1:** Comparison of BanglaMix with the closest existing Bengali-English and related code-mixed datasets.

Dataset	Scale	Token-level language ID	Bengali transliteration	Primary focus
Hindi-English & other Indian code-mixed LID corpora [[1], [2], [3], [4],^6^,^7^]	Typically small-to-medium (thousands of tweets/posts)	Yes - word-level LID, but for Hindi-English and other Indian pairs, not Bengali	No	Word-level language-identification shared tasks and corpora for non-Bengali Indian code-mixed pairs
DravidianCodeMix [8]; Malayalam-English sentiment corpus [10]	∼71,000 YouTube comments (Tamil-, Kannada-, Malayalam-English)	No - comment-level labels only	No	Sentiment and offensive-language classification for Dravidian code-mixed languages
BnSentMix [11]	20,000 comments (Facebook, YouTube, e-commerce)	No - comment-level sentiment labels only	No	Four-class sentiment classification for Bengali-English code-mixed text
BanglaTLit [12]	42.7k transliteration pairs (+245.7k pre-training corpus)	Not applicable - not annotated for code-mixing	Yes - Romanized-to-Bangla pairs, but not paired with naturalistic code-mixed social media comments or language tags	Back-transliteration benchmark for Romanized Bangla
BanglaMix (this work)	12,576 comments / 120,866 tokens	Yes - 5-way tags (BAN, ENG, ROM_BAN, OTHER, UNK) for every token	Yes - Bengali-script transliteration paired with every comment	Token- and comment-level annotated Bengali-English code-mixed YouTube comments, combining word-level LID, transliteration, and code-mix-type labels in a single resource

## 4 Data Description

The dataset is distributed as a single comma-separated values (CSV) file, dataset.csv, containing 12,576 rows (one row per comment) and 8 columns. Table 2 describes the fields in this file.

**Table 2:** Fields in dataset.csv.

Field	Type	Description
id	String	Unique alphanumeric identifier for each comment, consistent with the comment-ID format returned by the YouTube Data API. No usernames, channel names, or other personal identifiers are stored.
raw_text	String	Original, unprocessed text of the comment, in its original script (Bengali, Latin/Romanized, or mixed).
cleaned_text	String	Lightly preprocessed version of raw_text: leading @-mention tokens removed, and line breaks / repeated whitespace collapsed to single spaces. Otherwise unchanged from raw_text.
bengali_transliteration	String	Bengali-script (Unicode) transliteration of cleaned_text, produced during annotation.
token_labels	String	Whitespace-tokenized cleaned_text with each token tagged as token/LABEL, where LABEL is one of BAN, ENG, ROM_BAN, OTHER or UNK (see Table 5).
script_type	Categorical	Overall script composition of the comment: latin, bengali, mixed, or other.
code_mix_type	Categorical	Category describing which languages/scripts co-occur in the comment (8 categories; see Table 4).
likely_code_mixed	Boolean	True/False flag indicating whether the comment is likely to exhibit code-mixing.

Table 3 shows three illustrative, anonymised example rows from the dataset (comment identifiers shortened for display); these are reproduced only to illustrate the structure of the fields described in Table 2.

**Table 3:** Illustrative example records (abridged).

Table 4 summarises overall dataset size, uniqueness, missing-value, and duplication statistics, as reported by exploratory data analysis of the released file (Fig. 1).

**Table 4:** Dataset-level summary statistics.

Statistic	Value
Total records	12,576
Total columns	8
Unique comment IDs	12,576 (100%)
Unique raw_text values	12,576 (100%)
Unique bengali_transliteration values	12,420 (98.76%)
Missing values (all fields)	0 (0.00%) in every field
Duplicate comment IDs	0
Duplicate raw_text values	0
Duplicate bengali_transliteration values	156 (i.e., 156 distinct source comments happen to transliterate identically, most often short, formulaic comments such as greetings or single-word reactions)
Duplicate raw_text–bengali_transliteration pairs	0

**Fig. 1:**
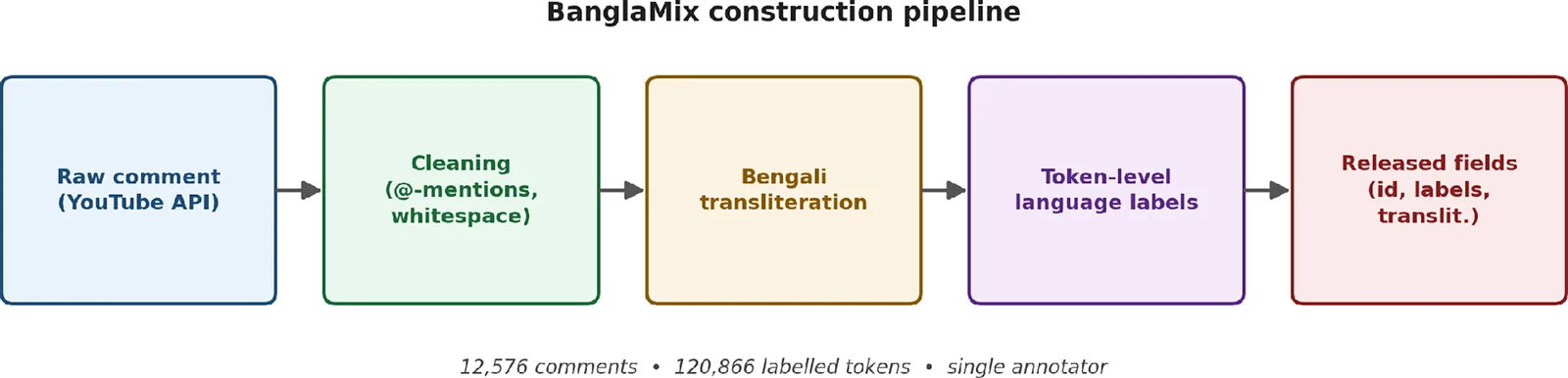
Overview of the BanglaMix construction pipeline: a raw comment retrieved via the YouTube Data API is cleaned, transliterated into Bengali script, and tagged at the token level, before the derived annotations are released.

Fig. 2 shows the distribution of the comment-level script_type field.

**Fig. 2:**
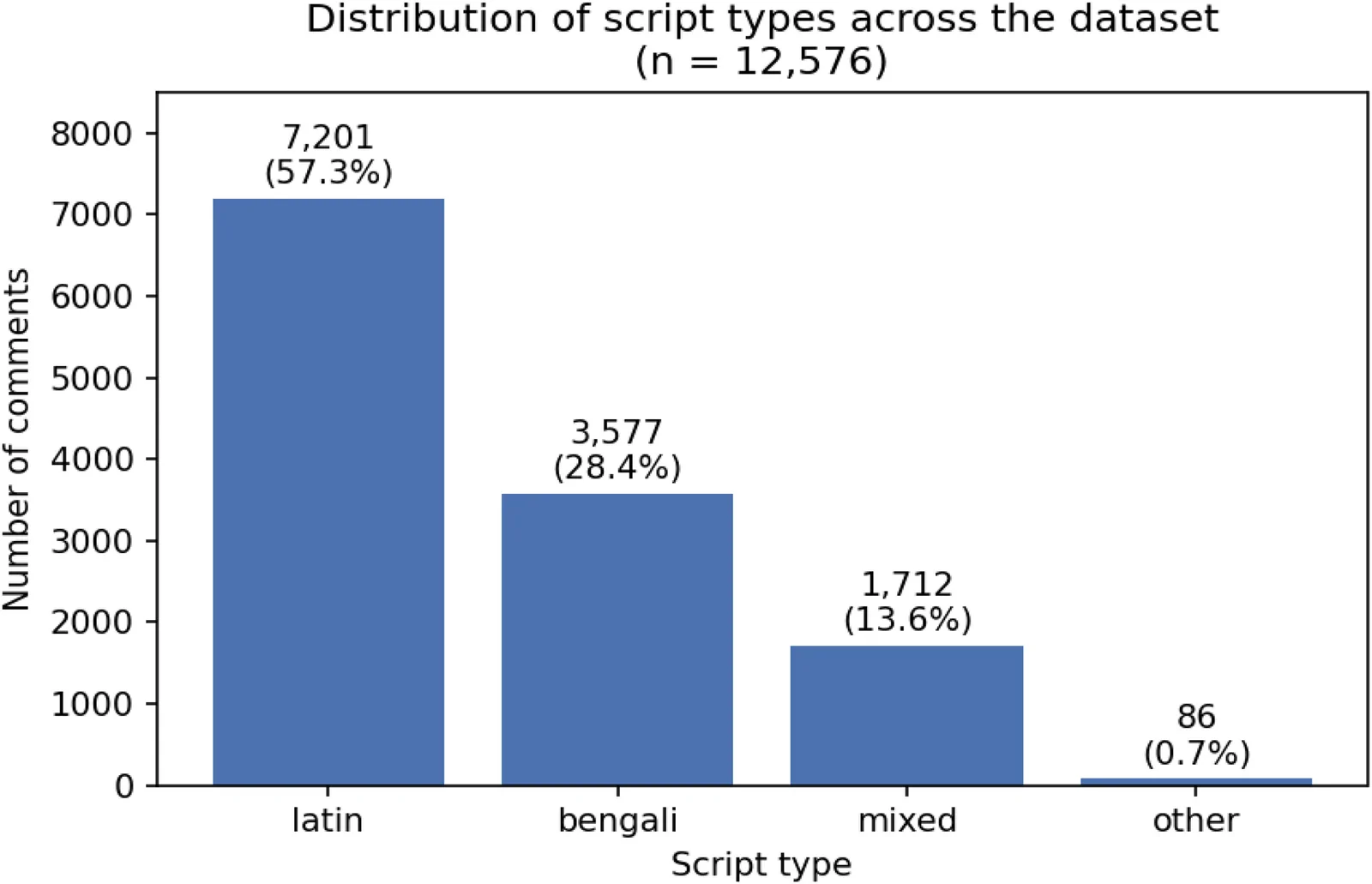
Distribution of the script_type field across all 12,576 comments.

Table 5a reports the same script_type distribution in tabular form.

**Table 5a:** script_type distribution.

script_type	Count	Percentage
latin	7201	57.26%
bengali	3577	28.44%
mixed	1712	13.61%
other	86	0.68%

Fig. 3 shows the distribution of the eight-category code_mix_type field.

**Fig. 3:**
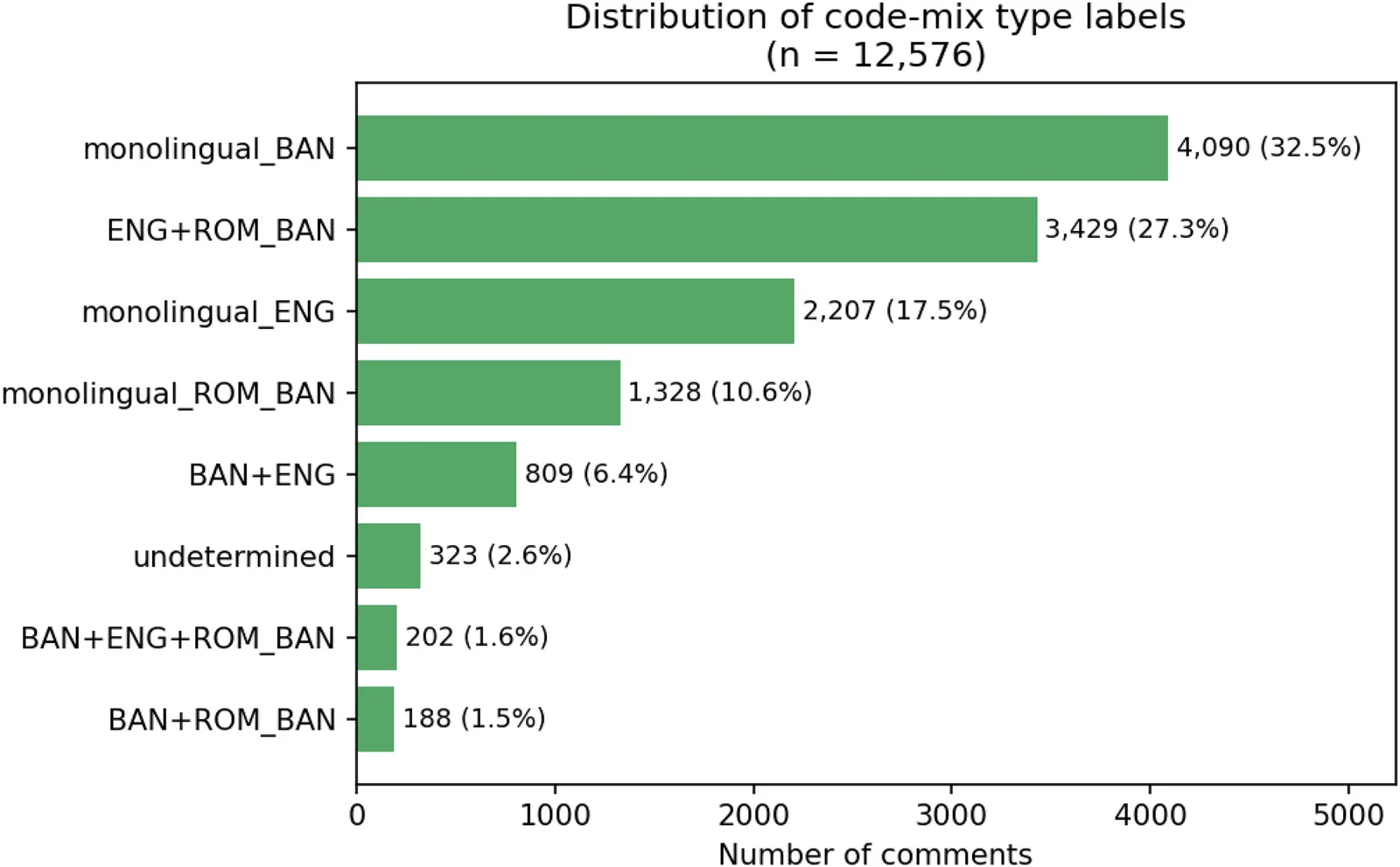
Distribution of the code_mix_type field across all 12,576 comments.

Table 5b reports the distribution of code_mix_type and of the likely_code_mixed boolean flag in tabular form.

**Table 5b:** code_mix_type and likely_code_mixed distributions.

code_mix_type	Count	Percentage
monolingual_BAN	4090	32.52%
ENG+ROM_BAN	3429	27.27%
monolingual_ENG	2207	17.55%
monolingual_ROM_BAN	1328	10.56%
BAN+ENG	809	6.43%
undetermined	323	2.57%
BAN+ENG+ROM_BAN	202	1.61%
BAN+ROM_BAN	188	1.49%
likely_code_mixed = False	7948	63.20%
likely_code_mixed = True	4628	36.80%

Fig. 4 shows the distribution of word-level language labels across all tokens in the token_labels field.

**Fig. 4:**
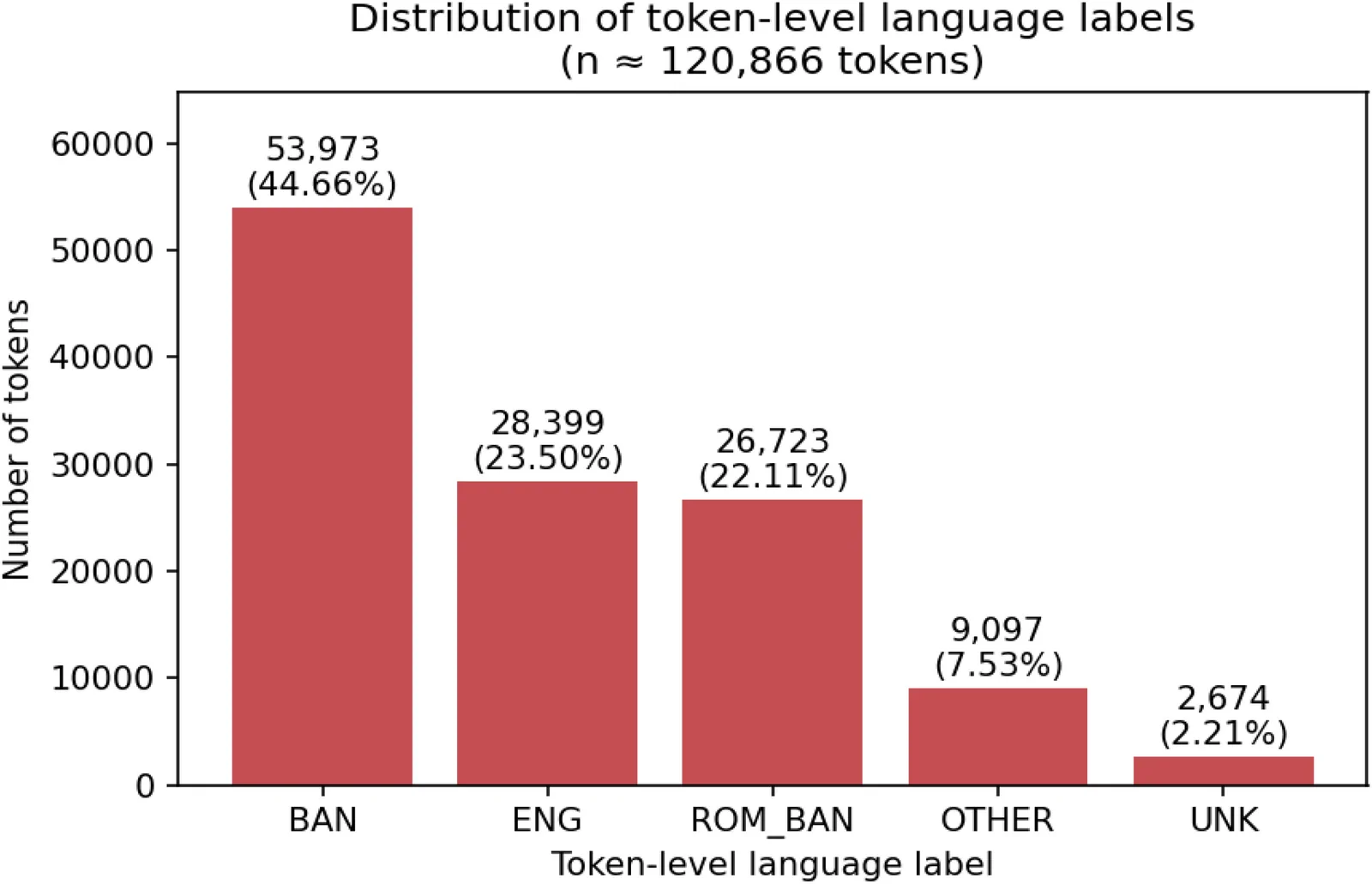
Distribution of token-level language labels (n = 120,866 labelled tokens).

Table 6 reports the same word-level language label distribution in tabular form.

**Table 6:** Token-level language label distribution.

Fig. 5 shows the length of the source (cleaned_text) field in words per comment.

**Fig. 5:**
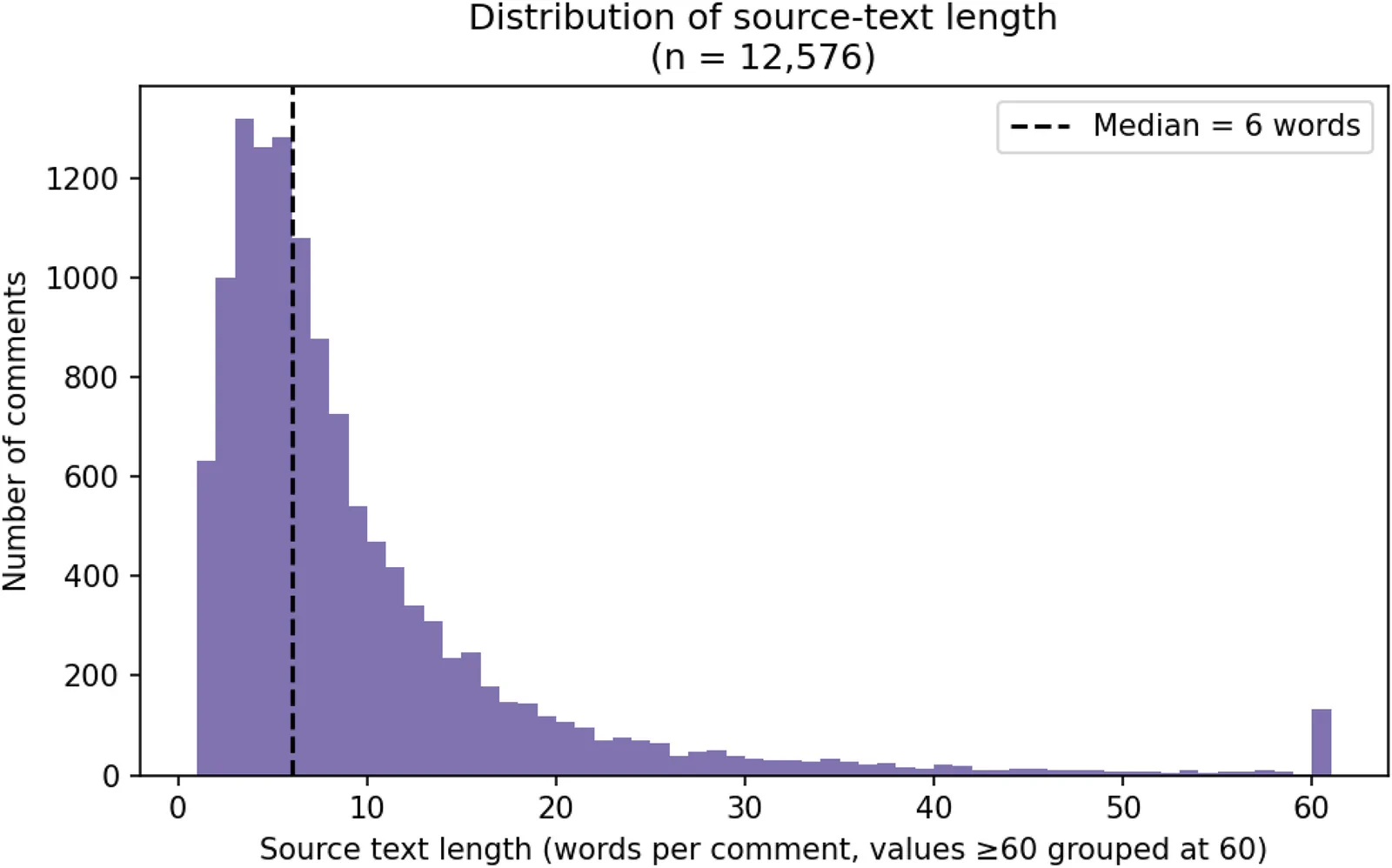
Distribution of source-text length in words per comment (values ≥ 60 grouped at 60 for readability); n = 12,576.

Table 7 reports text-length statistics in words and characters for both the source (cleaned_text) and target (bengali_transliteration) fields.

**Table 7:** Text-length statistics (source = cleaned_text; target = bengali_transliteration).

Measure	Mean	Median	SD	Min	Max
Source words	9.61	6.00	11.79	1	196
Target words	9.64	6.00	11.81	1	196
Source characters	43.44	28.00	55.10	2	1378
Target characters	43.42	28.00	54.85	1	1378
Bengali target characters (Bengali-script span only)	39.84	25.00	50.81	0	832
Latin source characters (Latin-script span only)	19.88	10.00	34.84	0	633

Finally, Table 8 reports summary statistics on the relationship between source (cleaned_text) and target (bengali_transliteration) sequence lengths. The ratios cluster tightly around 1.0 because transliteration here is a near one-to-one script transcription rather than a translation: each source token generally maps to one Bengali-script rendering of comparable length, so the operation adds or removes very little text overall.

**Table 8:** Source-target length relationship.

Measure	Value
Mean word-count ratio (target/source)	1.006
Median word-count ratio (target/source)	1.000
Mean character-count ratio (target/source)	1.010
Median character-count ratio (target/source)	1.000

### 4.1 Formal definition of comment-level code-mixing

The comment-level attributes introduced above (script_type, code_mix_type, and likely_code_mixed) are not independent judgements; each is computed deterministically from the token-level labels defined in Table 6. This subsection states those computations explicitly, because they define how the degree of code-mixing, that is, the code-mixing complexity, of each comment is evaluated. Let a comment c consist of an ordered sequence of n whitespace-delimited tokens, and let each token carry exactly one label from the five-label set

$$\ell_{i}\in Y=\left\{BAN,ENG,ROM\_BAN,OTHER,UNK\right\},i=1,\ldots ,n$$

For each of the three language- or script-bearing labels L ∈ {BAN, ENG, ROM_BAN}, the presence of that label anywhere in the comment is recorded by the indicator function

$$\delta_{L}\left(c\right)=\{1,if\;\exists i\in \left\{1,\ldots ,n\right\}:\ell_{i}=L0,otherwise,L\in \left\{BAN,ENG,ROM\_BAN\right\}$$

The set of distinct language and script categories that co-occur in the comment is then

$$\kappa \left(c\right)=\left\{L\in \left\{BAN,ENG,ROM\_BAN\right\}:\delta_{L}\left(c\right)=1\right\}$$

and the code-mixing complexity of the comment is the number of distinct categories it contains:

$$C\left(c\right)=\left|\kappa \left(c\right)\right|=\delta_{BAN}\left(c\right)+\delta_{ENG}\left(c\right)+\delta_{ROM\_BAN}\left(c\right)\in \left\{0,1,2,3\right\}$$

This quantity is 0 for undetermined comments, 1 for monolingual comments, 2 for comments that mix exactly two language/script categories, and 3 for comments that mix all three. The eight-valued code_mix_type category is a direct function of κ(c), assigned by the following rule:

$$\text{code}\_\text{mix}\_\text{type}\left(c\right)=\{\;\begin{array}{cc} \text{undetermined}, & \text{if}\;\kappa \left(c\right)=\emptyset \\ \text{monolingual}\_\text{BAN}, & \text{if}\;\kappa \left(c\right)=\left\{\text{BAN}\right\}\\ \text{monolingual}\_\text{ENG}, & \text{if}\;\kappa \left(c\right)=\left\{\text{ENG}\right\}\\ \text{monolingual}\_\text{ROM}\_\text{BAN}, & \text{if}\;\kappa \left(c\right)=\left\{\text{ROM}\_\text{BAN}\right\}\\ \text{BAN}\;+\;\text{ENG}, & \text{if}\;\kappa \left(c\right)=\left\{\text{BAN},\text{ENG}\right\}\\ \text{BAN}\;+\;\text{ROM}\_\text{BAN}, & \text{if}\;\kappa \left(c\right)=\left\{\text{BAN},\text{ROM}\_\text{BAN}\right\}\\ \text{ENG}\;+\;\text{ROM}\_\text{BAN}, & \text{if}\;\kappa \left(c\right)=\left\{\text{ENG},\text{ROM}\_\text{BAN}\right\}\\ \text{BAN}\;+\;\text{ENG}\;+\;\text{ROM}\_\text{BAN}, & \text{if}\;\kappa \left(c\right)=\left\{\text{BAN},\text{ENG},\text{ROM}\_\text{BAN}\right\} \end{array}$$

A comment is flagged as likely code-mixed exactly when it contains two or more distinct language/script categories, that is, when its code-mixing complexity is at least two:

likely_code_mixed(c)={1,ifC(c)≥20,ifC(c)≤1

Applied to the released data, this rule yields likely_code_mixed = True for exactly the four mixed categories ENG+ROM_BAN, BAN+ENG, BAN+ROM_BAN, and BAN+ENG+ROM_BAN (4628 comments; 36.80%) and False for the remaining four categories (7948 comments; 63.20%), matching the distribution reported in Table 5b.

The script_type attribute is defined separately, at the level of writing systems rather than token labels. Because OTHER and UNK tokens carry no definite language, script composition is evaluated over the characters of the cleaned comment string s(c). Let B denote the Bengali Unicode block (U + 0980–U + 09FF) and let A denote the set of Latin letters (A–Z, a–z); the two script indicators are

b(c)=1[B∩s(c)≠∅],l(c)=1[A∩s(c)≠∅]

These indicators yield the four-valued script_type category:

$$\text{script}\_\text{type}\left(c\right)=\{\;\begin{array}{cc} \text{mixed}, & \text{if}\;b\left(c\right)=1\;\text{and}\;l\left(c\right)=1\\ \text{bengali}, & \text{if}\;b\left(c\right)=1\;\text{and}\;l\left(c\right)=0\\ \text{latin}, & \text{if}\;b\left(c\right)=0\;\text{and}\;l\left(c\right)=1\\ \text{other}, & \text{if}\;b\left(c\right)=0\;\text{and}\;l\left(c\right)=0 \end{array}$$

A comment whose text contains only Latin characters (English words or Romanized Bengali) is therefore labelled latin; one containing only Bengali-script characters is labelled bengali; one containing characters from both scripts is labelled mixed; and one containing neither, such as a comment composed only of digits, punctuation, or symbols, is labelled other. The reported script_type distribution in Table 5a (latin 57.26%, bengali 28.44%, mixed 13.61%, other 0.68%) follows directly from this rule.

## 5 Experimental Design, Materials and Methods

### 5.1 Data collection

Comments were collected from publicly available YouTube videos using the YouTube Data API v3 (googleapiclient, Python), via a purpose-built collection script. The script supported two acquisition modes: (i) a channel-based mode, in which comments were pulled from the upload history of known Bangla/Bangladesh-related creators by resolving each channel’s uploads playlist (playlistItems.list) and then paging through top-level comments and replies for each video (commentThreads.list); and (ii) a search-based discovery mode (search.list), which queried a fixed set of seed terms in Bengali script, Roman-transliterated Bengali, and English, restricted to region code “BD” and Bengali relevance language. The topic categories named in this article (music, natok/drama, news, cricket, comedy, vlogs, cooking, cinema, religious content, technology, education, gaming, travel, and discussion/interview content) are the actual seed-term categories used to construct the search queries, not merely illustrative examples; the complete set of 56 seed queries used in the search-based mode is released alongside the dataset in the Zenodo record (see Data Accessibility), whereas the specific channel list used in the channel-based mode was not retained in a form that could be recovered for this article (see Limitations).

Both top-level comments and their replies were retrieved (commentThreads.list with part=“snippet, replies”); comments authored by the video’s own channel/uploader were excluded at collection time, as these were predominantly promotional rather than organic user text. Comment order alternated between “relevance” and “time” across videos: “relevance” surfaces highly engaged comments that reflect what other viewers found notable, while “time” surfaces recent comments regardless of engagement, so alternating between the two reduces the bias toward high-like-count pinned or promotional comments that ordering by relevance alone would introduce, without discarding relevance-ranked content entirely. A local state file tracked already-processed video IDs so that repeated runs (needed to work within the API’s daily quota) did not reprocess the same videos.

The dataset contains 12,576 unique comment records with 12,576 unique comment identifiers and 12,576 unique raw_text values (i.e., no two records share identical raw comment text). The collection script does not perform explicit text-level deduplication of comments; its only duplicate-avoidance mechanism operates at the video level, via a resumable state file that records already-processed video IDs so that a video’s comments are not re-fetched across multiple runs. The absence of duplicate raw_text values in the collected data therefore most likely reflects this video-level dedup (each comment is fetched at most once) combined with the low prior probability of two different users independently posting identical free-text comments, rather than any deliberate text-similarity filtering.

The script applies two content-level exclusion rules at collection time: comments and replies authored by the video’s own channel/uploader are dropped (identified by matching the commenter’s display name against the channel title), and paging for a given video stops once a per-video comment cap (max_comments_per_video, default 300) is reached. No minimum/maximum comment-length filter, spam/bot filter, or language pre-filter is applied in the script; any such filtering of the 12,576 collected records occurred after collection, if at all, and is not captured in the script.

### 5.2 Preprocessing

Each raw comment (raw_text) was lightly cleaned to produce cleaned_text. An earlier version of the collected data included 145 records whose raw_text began with a leading @-mention addressing another commenter; rather than only stripping the mention from cleaned_text, these records were removed entirely from the released dataset.csv, so no raw_text value in the released file retains a username or other public display-name reference. Comparison of the two fields across the released 12,576 records indicates that the remaining cleaning consisted of collapsing line breaks and repeated whitespace into single spaces (affecting 1718 of 12,576 records, 13.66%). No hyperlinks were present in raw_text (0 records). No other systematic transformation (e.g., lower-casing, punctuation removal, spelling normalisation) was applied between raw_text and cleaned_text.

### 5.3 Annotation procedure

All annotations in this dataset, the Bengali-script transliteration, the word-level token_labels, and the comment-level script_type, code_mix_type, and likely_code_mixed tags were produced by a single annotator (the author). To provide an objective empirical benchmark for the reliability of these labels, a second qualified bilingual annotator independently re-annotated a random, stratified sub-sample of 500 comments; the resulting token- and comment-level inter-annotator agreement statistics are reported in Inter-annotator reliability below and summarised in Limitations.

Based on consistent patterns observed across the released token_labels field, the five occurring word-level labels appear to correspond to the following categories: BAN denotes a Bengali-language word written in Bengali (Unicode) script; ROM_BAN denotes a Bengali-language word written in Romanized (Latin-script) form; ENG denotes an English-language word; OTHER denotes punctuation, symbols, emoji, or other non-linguistic tokens; and UNK denotes a token of undetermined language. These category interpretations are inferred from observed labelling patterns in the data rather than reproduced from a written annotation manual, which was not among the materials provided for this article.

### 5.4 Annotation guidelines

Each whitespace-delimited token in cleaned_text was assigned one of five labels: BAN, ENG, ROM_BAN, OTHER, or UNK. Table 6 (above) reports, for each label, its definition, the decision rule applied during annotation, a worked example, and its distribution across all 120,866 labelled tokens.

### 5.5 Inter-annotator reliability

To provide the objective empirical benchmark for annotation reliability requested in the review, a second qualified bilingual annotator, working independently and without access to the first annotator's (the author's) labels, re-annotated a random, stratified sub-sample of 500 comments (4.0% of the 12,576-comment corpus), assigning both word-level token_labels and the comment-level code_mix_type category. Token-level agreement was scored on the tokens that could be aligned between the two annotations (4857 tokens from the first annotator and 4883 from the second; 4644 tokens aligned and scored, 95.6% alignment coverage relative to the first annotator); comment-level agreement was scored on all 500 comments. For both levels, observed agreement (P_o), chance-corrected Cohen's kappa, and per-label/per-category precision, recall, and F1 (relative to the first annotator's labels as reference) were computed, together with 95% confidence intervals from a comment-clustered bootstrap (2000 resamples, seed 20,240,517).

Token-level agreement was substantial: P_o = 0.8305, expected agreement P_e = 0.3361, Cohen's kappa = 0.7448 (95% CI [0.7163, 0.7744]), corresponding to the ``substantial'' band of the Landis and Koch interpretation scale [14] (Table 9a). Per-label performance (Table 9b) was highest for BAN and OTHER tokens (F1 = 0.98 and 0.79) and lowest for ROM_BAN and UNK. Disagreement was concentrated rather than uniform: 545 of 1098 first-annotator ROM_BAN tokens were labelled ENG by the second annotator, and all 102 first-annotator UNK tokens were relabelled as BAN, ENG, or ROM_BAN by the second annotator, i.e., the second annotator used the UNK category far more sparingly. Both patterns point to a genuine, hard-to-eliminate judgement boundary between Romanized Bengali and English for short, ambiguous, or borrowed tokens (numerals, interjections, names, onomatopoeia) rather than to careless labelling.

**Table 9a:** Token-level inter-annotator agreement summary (500-comment sub-sample, second annotator vs. first annotator).

Metric	Value
Tokens, first annotator	4857
Tokens, second annotator	4883
Aligned tokens (scored)	4644
Alignment coverage (rel. to first annotator)	0.9561
Observed agreement ($P_{o}$)	0.8305
Expected agreement ($P_{e}$)	0.3361
Cohen's kappa	0.7448
95% CI (bootstrap)	[0.7163, 0.7744]
Interpretation [14]	Substantial

**Table 9b:** Token-level per-label agreement (support and precision/recall/F1 of the second annotator's labels relative to the first annotator's labels).

Label	Support	Precision	Recall	F1
BAN	2052	0.9693	0.9990	0.9839
ENG	1229	0.6561	0.9455	0.7747
OTHER	163	0.9907	0.6564	0.7897
ROM_BAN	1098	0.8277	0.4900	0.6156
UNK	102	0.0000	0.0000	0.0000

Comment-level code_mix_type agreement was likewise substantial: P_o = 0.7480, P_e = 0.2465, Cohen's kappa = 0.6656 (95% CI [0.6192, 0.7109]), macro F1 = 0.5076 (Table 10a). Table 10b shows that the two largest single categories, monolingual_BAN (F1 = 0.96) and ENG+ROM_BAN (F1 = 0.74), were labelled reliably, while the rarer three-way and pairwise mixed categories (BAN+ROM_BAN; BAN+ENG+ROM_BAN) and the monolingual_ROM_BAN and undetermined categories were harder to reproduce; most monolingual_ROM_BAN comments in the first annotator's labels were re-labelled ENG+ROM_BAN by the second annotator, and all 13 undetermined comments were assigned a definite category by the second annotator. This mirrors the token-level finding that Romanized Bengali/English boundaries, and the residual UNK/undetermined categories, are the main source of disagreement, whereas the core monolingual and pairwise categories are labelled consistently across annotators.

**Table 10a:** Comment-level (code_mix_type) inter-annotator agreement summary (500-comment sub-sample).

Metric	Value
Comments	500
Observed agreement ($P_{o}$)	0.7480
Expected agreement ($P_{e}$)	0.2465
Cohen's kappa	0.6656
95% CI (bootstrap)	[0.6192, 0.7109]
Macro F1	0.5076
Interpretation [14]	Substantial

**Table 10b:** Comment-level per-category agreement (support and precision/recall/F1 of the second annotator's labels relative to the first annotator's labels).

Category	Support	Precision	Recall	F1
BAN+ENG	32	0.7692	0.6250	0.6897
BAN+ENG+ROM_BAN	8	0.4286	0.7500	0.5455
BAN+ROM_BAN	7	0.2000	0.1429	0.1667
ENG+ROM_BAN	136	0.6519	0.8676	0.7445
monolingual_BAN	163	0.9515	0.9632	0.9573
monolingual_ENG	88	0.6562	0.7159	0.6848
monolingual_ROM_BAN	53	0.6923	0.1698	0.2727
undetermined	13	0.0000	0.0000	0.0000

Both the token-level (kappa = 0.7448) and comment-level (kappa = 0.6656) agreement statistics fall in the ``substantial'' band of the Landis and Koch scale [14], providing the objective, quantitative reliability benchmark that was absent from the original submission. The released dataset's silver-standard labels should therefore be read as reliable at the level of the coarse, high-support categories (BAN, ENG, monolingual_BAN, ENG+ROM_BAN) and as noisier for the rarer three-way mixed categories and for the residual UNK/undetermined labels. The full second-annotator labels for this 500-comment sub-sample, the token-alignment procedure, and the bootstrap code used to compute the statistics above are released as supplementary material accompanying this article, so that the reliability analysis can be independently reproduced or extended to a larger sub-sample.

### 5.6 Quality checks

Exploratory data analysis was performed on the released dataset.csv without modifying the original file, covering dataset size, script-type and code-mix-type distributions, token-level label distributions, text-length statistics, missing-value counts, duplicate-record counts, and source-target length-ratio statistics. All statistics reported in the Data Description section above were computed from this analysis; missing values were found in none of the eight fields, and 156 comments shared an identical bengali_transliteration value with at least one other comment despite having distinct raw_text and comment IDs.

## Limitations

Annotation (Bengali transliteration, word-level language labels, script_type, code_mix_type, and likely_code_mixed) was performed by a single primary annotator. To address this, a second qualified bilingual annotator independently re-annotated a random, stratified sub-sample of 500 comments (4.0% of the corpus); the resulting inter-annotator agreement was substantial at both the token level (Cohen’s kappa = 0.7448, 95% CI [0.7163, 0.7744]) and the comment level (code_mix_type; Cohen’s kappa = 0.6656, 95% CI [0.6192, 0.7109]) (see Inter-annotator reliability). This provides an objective empirical benchmark for annotation consistency where none was previously available, but it does not amount to full double annotation of the corpus: the remaining 96.0% of comments carry only the first annotator’s labels. Because this dataset’s value rests entirely on its labels rather than on raw text alone, users should treat all annotation layers as silver-standard rather than gold-standard: they are suitable for training and for large-scale distributional analysis, but conclusions requiring guaranteed per-instance label correctness (e.g., using this dataset alone as a held-out evaluation benchmark for leaderboard-style comparison) should be corroborated with independently annotated data before being treated as authoritative, particularly for the rarer three-way mixed, Romanized-Bengali, and undetermined/UNK categories where the validation study found the lowest agreement. Extending double annotation beyond this 500-comment sub-sample is noted here as a priority for future work on this resource.

The general data-collection procedure (channel-based and search-based acquisition via the YouTube Data API, described under Experimental Design, Materials and Methods) is documented via the collection script’s logic and the seed-term topic categories used. The specific channel list used in the channel-based collection mode and the exact collection date range were not retained in a form that could be recovered for this article, which limits exact reproducibility of the channel-based portion of the sampling frame. The search-based portion is fully reproducible: all 56 seed queries used in the search-based collection mode are released alongside the dataset in the Zenodo record (see Data Accessibility).

**Data overlap with existing datasets.** Unlike the annotation and reproducibility limitations above, this limitation concerns the relationship between BanglaMix and previously published resources. The comments in BanglaMix were collected de novo for this study directly from YouTube through the YouTube Data API; no comment text from any existing corpus was reused, seeded, or merged into the collection. However, several comparable Bengali-English resources have already been published, most notably BanglaTLit [12] and BnSentMix [11], together with the other Bengali-related datasets cited in the Background, and the authors did not perform a systematic, record-level overlap analysis comparing BanglaMix against those resources. Because the datasets were constructed from different platforms, time windows, and sampling frames, and because the public archive stores only the derived annotations and platform comment IDs (raw comment text being re-fetched live from YouTube; see Data Accessibility), the extent of any incidental overlap cannot be stated with certainty. The comparison in Table 1 is therefore a feature-level comparison, not an overlap or deduplication analysis, and BanglaMix should not be assumed to be provably disjoint from BanglaTLit, BnSentMix, or the other cited corpora. Quantifying record-level overlap against these datasets is noted as future work.

The public YouTube API Services Terms of Service impose data-retention and third-party-access restrictions on API-sourced data. This dataset’s public release has been restructured accordingly: the openly and indefinitely distributed archive contains only the derived annotation fields (transliteration, token labels, script type, code-mix type, likely-code-mixed flag) and the comment ID, while raw_text and cleaned_text are obtained on demand via an accompanying re-hydration script rather than persisted in the archive (see Data Accessibility and Ethics Statement).

The code_mix_type categories are strongly imbalanced, ranging from 32.52% (monolingual_BAN) to 1.49% (BAN+ROM_BAN) of comments. This is most consequential for downstream supervised modelling tasks that require reliable performance on minority categories. For example, BAN+ROM_BAN and BAN+ENG+ROM_BAN (1.49% and 1.61% of comments respectively) are too sparse in this release alone to support robust per-category evaluation, and users targeting these categories specifically should consider augmenting with additional data or using stratified evaluation protocols.

An earlier version of this dataset retained a small number of raw_text values with a leading @-mention referencing another commenter’s public display name; the 145 affected records were removed prior to release, and no raw_text value in the collected dataset retains a username, channel name, or other display-name reference.

The id field is consistent with the comment-ID format returned by the YouTube Data API and is very likely either the original platform comment ID or a value trivially derivable from it. This means the ID is not a synthetic anonymisation token: a user of this dataset could, in principle, query the YouTube Data API with a given ID to look up metadata associated with the original comment (e.g., the commenter’s public display name, if still available) that is not itself stored in this release. This does not necessarily make the release inappropriate. Platform-native IDs are common practice in social media NLP datasets, but it means the practical privacy guarantee is that no personal identifier is directly stored, not that the record is fully de-identified from its source platform (see Ethics Statement).

The dataset reflects comments on a specific, incompletely documented subset of content and commenters who chose to comment publicly in Bengali, English, or a mixture of the two; it should not be assumed to be representative of Bengali speakers, social media users generally, or code-mixing patterns more broadly.

## Ethics Statement

This work involved data collected from a social media platform (YouTube). The dataset does not contain directly stored personal identifiers such as usernames, channel names, or user IDs; each comment is represented by an alphanumeric comment ID together with its annotation layers. This ID is consistent with the format returned by the YouTube Data API and is therefore traceable to the source platform: it is not a synthetic, fully de-identified token, and a user of the dataset could in principle use it to query the YouTube Data API for metadata about the original comment not stored in this release (see Limitations). Records whose raw_text had retained a public @-mention referencing another commenter’s display name (145 records) were removed prior to release.

Comments were retrieved via the YouTube Data API v3, whose governing YouTube API Services Terms of Service (ToS) impose restrictions directly relevant to this dataset’s public release. In particular, the ToS generally limits retention of API-sourced data to 30 calendar days unless the data falls under specific exceptions, and restricts making API Data available to third parties outside the bounds of an authorised API Client. To resolve this tension, the public archive on Zenodo has been restructured to distribute only the derived annotation layers (bengali_transliteration, token_labels, script_type, code_mix_type, likely_code_mixed) together with each comment’s ID indefinitely; raw_text and cleaned_text are not persisted in the archive and are instead obtainable on demand via the accompanying rehydrate.py script, which re-fetches this text live from the YouTube Data API using a downstream user’s own API credentials. This mirrors common practice among other social-media NLP datasets subject to equivalent platform ToS restrictions (e.g., ID-plus-re-hydration release patterns used for Twitter/X-derived corpora).

## CRediT Author Statement

**Sabbir Mahmud Afridi:** Conceptualization, Data curation, Formal analysis, Investigation, Methodology, Resources, Software, Validation, Visualization, Writing – original draft, Writing – review &amp; editing. **Abir Chakraborty Partha:** Writing – review &amp; editing. **Md. Mosaddek Khan:** Supervision.

## AI Use Disclosure

The dataset collection, annotation, and all reported statistics were produced by the author without AI assistance. No generative AI tool was used to create, modify, or label any record in the released dataset itself. Generative AI tools were used during the preparation of the manuscript for language editing, improving clarity, and assisting with the organization and refinement of the written text. The author reviewed and verified all AI-assisted content and remains fully responsible for the final manuscript and its contents.

## Appendix Supplementary materials

Image, application 1
